# Case Report: Compound heterozygous familial hypercholesterolemia in a pediatric patient with multiple cutaneous xanthomas

**DOI:** 10.3389/fped.2026.1752339

**Published:** 2026-03-31

**Authors:** Qian Yang, Lihong Jiang, Xinyi Wei, Xinyang Lv, Yue Zhao, Geli Liu

**Affiliations:** Department of Pediatrics, Tianjin Medical University General Hospital, Tianjin, China

**Keywords:** familial hypercholesterolemia, lipoprotein apheresis, low-density lipoprotein cholesterol, low-density lipoprotein receptor, statins, xanthomas

## Abstract

Familial hypercholesterolemia (FH) is an inherited disorder of lipid metabolism characterized by markedly elevated plasma low-density lipoprotein cholesterol (LDL-C) levels, formation of xanthomas, and early-onset atherosclerotic cardiovascular disease (ASCVD). This report describes a rare pediatric case of compound heterozygous FH (cHeFH), in which two mutations were identified in the low-density lipoprotein receptor (*LDLR*) gene through whole-exome sequencing (WES), including c.682G > A and the rarely reported c.1187–10G > A variant. This case provides key insights into the association between these mutations, the clinical phenotype, and treatment response.

## Introduction

Familial hypercholesterolemia (FH) is a rare yet potentially life-threatening disorder, in which cutaneous manifestations may constitute the only clinical indication of the disease (1). Despite a global pediatric prevalence of approximately 1 in 364 (2), as many as 95% of children with FH remain undiagnosed and untreated worldwide (3). FH is categorized into homozygous FH (HoFH) and heterozygous FH (HeFH) forms according to the underlying genetic variants. HoFH is rare, with an estimated prevalence ranging from approximately 1 in 250,000 to 1 in 360,000 individuals (4). HoFH can be further classified into true homozygous, compound heterozygous(cHeFH), and double heterozygous forms depending on the mutation status of the affected alleles (5). HoFH is a severe inherited metabolic disorder characterized by the accelerated development of cardiovascular disease beginning in early life. Without treatment, the majority of patients with HoFH develop atherosclerotic cardiovascular disease (ASCVD) before 18 years of age and face a markedly increased risk of fatal coronary events by 31 years of age (4). Therefore, early diagnosis and intervention are crucial for the effective management of FH. This case report presents diagnostic and therapeutic insights from a rare case of cHeFH resulting from mutations in the low-density lipoprotein receptor (*LDLR*) gene.

## Case report

A 2-year-2-month-old girl presented to the Department of Pediatrics at Tianjin Medical University General Hospital with a one-year history of xanthomas. The lesions initially appeared as a rash with yellowish deposits around both ankles. Over time, they gradually enlarged to resemble rice grains and beans, and became scattered across the limbs and buttocks. Initial laboratory tests revealed a markedly elevated total cholesterol (TC) level of 22.5 mmol/L. The patient was born at term via cesarean section as the second child of a second pregnancy to non-consanguineous parents. Her growth and developmental milestones were appropriate for her age, and she had no previous history of routine serum lipid monitoring. There was no family history of similar dermatological findings, dyslipidemia, premature coronary heart disease, diabetes mellitus, or hypertension in her parents or older sibling.

**Figure 1:**
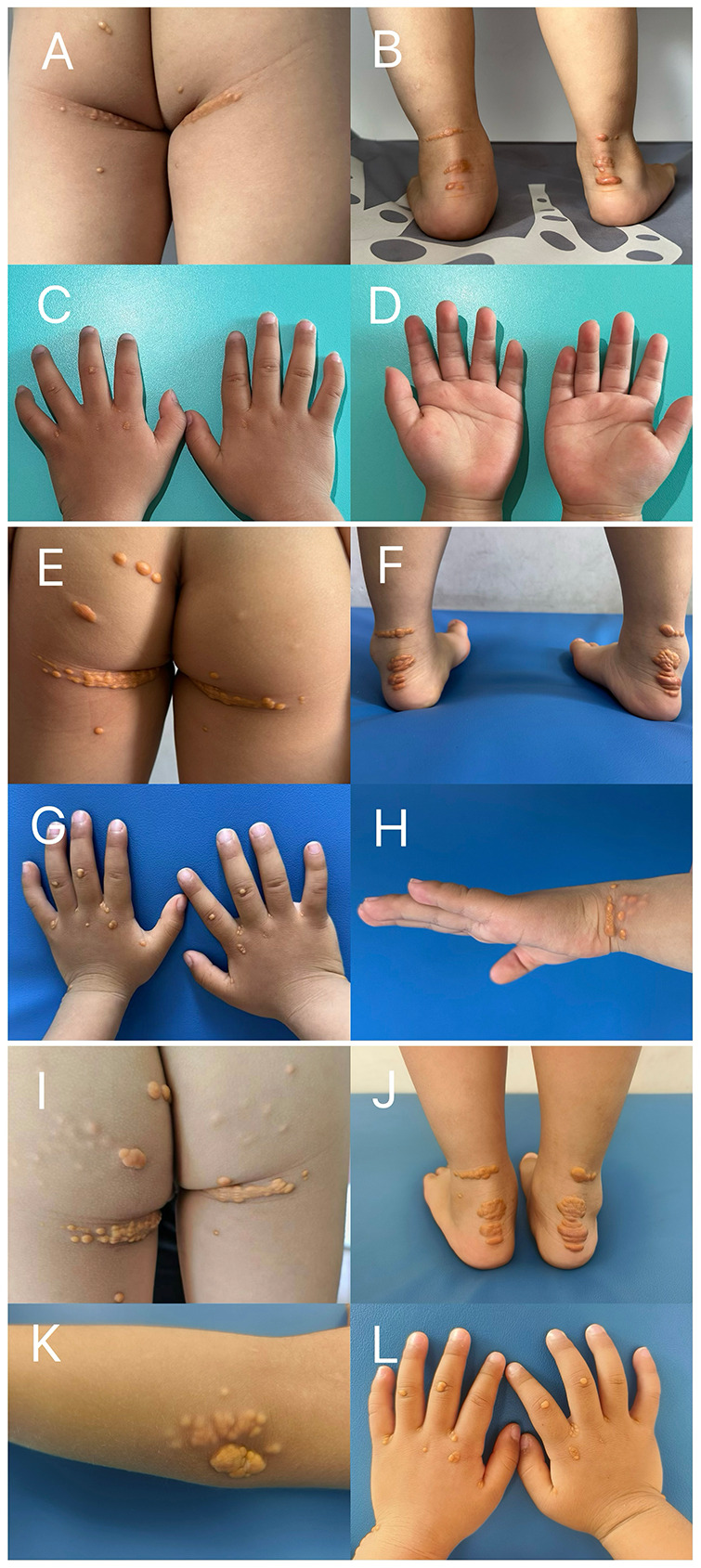
Progression of cutaneous xanthomas. Images depict xanthomas on the **(A,E,I)** buttocks, **(B,F,J)** bilateral Achilles tendons, **(C,D,G,L)** dorsal and ventral surfaces of the hands, **(H)** ulnar border of the left hand, and **(K)** left elbow. Images **(A–D)**, captured on October 23, 2024, show the initial extent of the lesions. Images **(E–H)**, captured on April 23, 2025, demonstrate a significant increase in the size and distribution of xanthomas compared with image **(A–D)** obtained six months prior. Images **(I–L)**, captured on October 20, 2025, reveal further progression of the xanthomas compared with the previous time points **(A–D)** and **(E–H)**.

Physical examination was performed, and the patient's height and weight were determined to be 87 cm and 12 kg, respectively, both within the 25th–50th percentile for age. The patient exhibited normal physical development and was alert and oriented. Multiple yellowish-brown nodular papules of varying sizes were observed on the buttocks and both ankles, slightly elevated above the skin surface, and locally arranged in linear patterns. The intervening skin appeared normal and no ulceration was noted (Figure 1). No significant abnormalities were detected on cardiac or pulmonary auscultation. Abdominal examination revealed that the abdomen was soft and non-distended, with no palpable hepatosplenomegaly below the costal margin, and bowel sounds were present. Examination of the musculoskeletal system revealed normal spinal alignment and unrestricted limb mobility. The external genitalia exhibited normal development, consistent with the patient's age.

Laboratory investigations, including assessment of complete blood count, hepatic and renal function, cardiac enzymes, fasting glucose and insulin, thyroid function, and urinalysis, revealed that all the parameters were within normal limits. However, lipid profile analysis revealed that several lipid fractions were elevated, including total cholesterol (TC): 21.5 mmol/L (normal range: 3.59–5.17 mmol/L); triglycerides (TG): 1.71 mmol/L (normal range: 0.57–1.71 mmol/L); high-density lipoprotein cholesterol (HDL-C): 0.81 mmol/L (normal range: 0.80–2.20 mmol/L); LDL-C: 17.12 mmol/L (normal range: 1.33 mmol/L–3.36 mmol/L); apolipoprotein A1: 0.6 g/dL (normal range: 1.2–1.8 g/L); apolipoprotein B: 4.15 g/L (normal reference range: 0.60–1.14 g/L); and lipoprotein(a): 433.2 mg/L (normal reference range: 0–300 mg/L). No significant abnormalities were detected on ultrasound examination of the liver, spleen, and kidneys, or on echocardiography. Skin CT revealed that the epidermis at the lesion sites was largely normal, with a poorly defined dermoepidermal junction. The superficial dermis displayed dense infiltration by numerous round cells with moderate- to high-refractive indices, some of which were fused into a ring-like pattern, accompanied by scattered inflammatory cell infiltration. Dermoscopy of the lesions revealed numerous large, yellowish-white papules with a homogeneous appearance.

**Figure 2:**
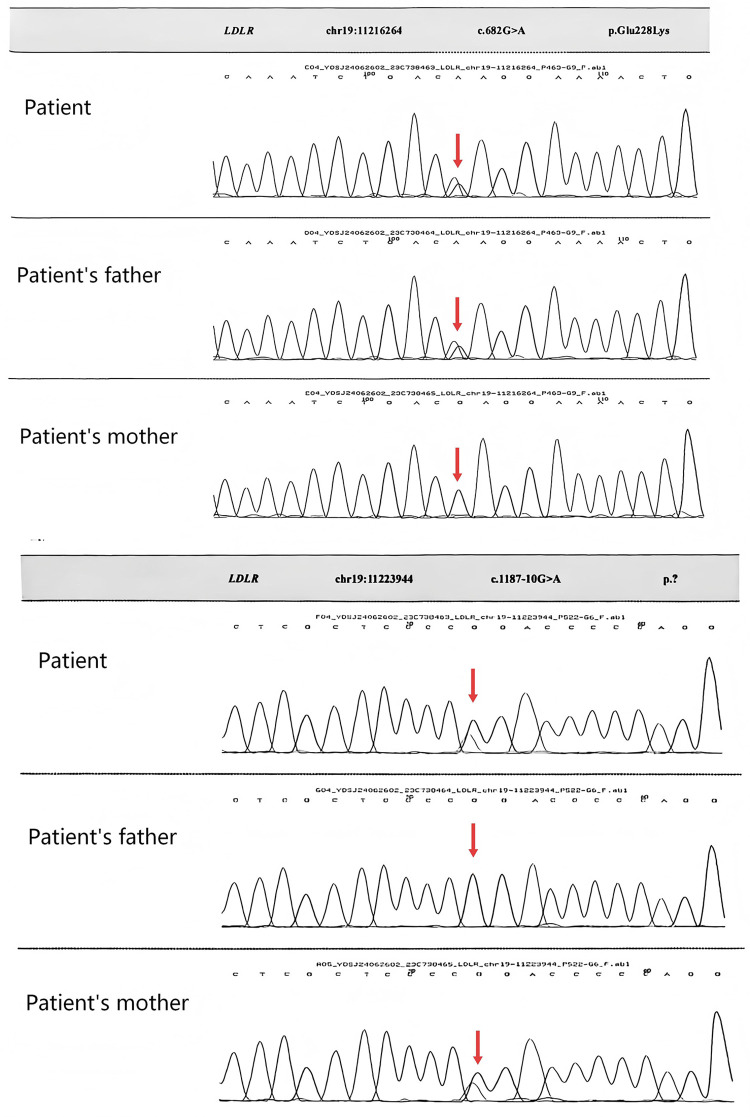
Sequencing map of the *LDLR* gene in the patient and her parents, with arrows indicating the mutation sites.

Whole-exome sequencing (WES) was performed after obtaining informed consent from the patient's family, and the findings were subsequently validated by Sanger sequencing to confirm a diagnosis of FH (Table 1). Analysis of the sequencing data revealed that the proband harbored a compound heterozygous mutation in the *LDLR* gene (Table 1). The c.682G > A mutation was identified in exon 4 of the *LDLR* gene. According to the ACMG guidelines, this variant was classified as pathogenic. Sanger sequencing further confirmed that this mutation was inherited paternally (Figure 2). We additionally identified a heterozygous c.1187-10G > A mutation in intron 8 of the *LDLR* gene. According to the ACMG guidelines, this variant was classified as likely pathogenic. Sanger sequencing confirmed that this mutation was inherited maternally, forming a compound heterozygous genotype with the previously described variant (Figure 2).

**Table 1:** Identified genetic variants and their predicted pathogenicity.

Variant	SIFT	PolyPhen-2	MutationTaster	ACMG classification	ACMG criteria
c.682G > A (p. Glu228Lys)	Harmful (0.001)	Potentially harmful (0.998)	Pathogenic (1)	Pathogenic	PS4 + PM1 + PM2_Supporting + PM5_Strong + PP3_Strong
c.1187-10G > A	-	-	-	Likely pathogenic	PS4 + PM2_Supporting + PP3

Following a diagnosis of FH, patients are generally advised to implement dietary modifications and adopt healthier lifestyle practices. Although the patient was only 2 years old, lipid-lowering medication was initiated following consultation with the family. Statin therapy was initiated at 2 years and 3 months of age (September 2024) with oral rosuvastatin at a dose of 2.5 mg/day. The lipid levels were monitored monthly, and the dose of rosuvastatin was gradually increased to 20 mg/day. After five months of oral rosuvastatin therapy, ezetimibe was added at a dose of 10 mg/day owing to the suboptimal reduction in TC and the exacerbation of xanthomas (Figure 1). During this period, liver function tests and assessment of creatine kinase and its isoenzymes revealed that the parameters remained largely within normal limits. Lipid panel analysis at the 3-year-4-month follow-up demonstrated that the levels of TC and LDL-C were 15.81 mmol/L and 12.28 mmol/L, respectively, substantially exceeding the established therapeutic targets (Table 2). Additionally, the xanthomas had rapidly progressed during this interval, indicating a suboptimal response to treatment (Figure 1). The patient's family members expressed considerable distress regarding the clinical situation. We therefore discussed the initiation of lipoprotein apheresis (LA) as a therapeutic option. However, due to time constraints, financial considerations, and logistical challenges, the family remains hesitant to proceed at this time.

**Table 2:** Drug treatment regimens and corresponding blood lipid levels in the patient.

Date	TC (mmol/L)	TG (mmol/L)	HDL-C (mmol/L)	LDL-C (mmol/L)	APO-A1 (g/L)	APO-B (g/L)	Lpa (mg/L)	Treatment
14th August 2024	22.5	1.54	-	-	-	-	-	Diet + exercise
25th September 2024	21.5	1.71	0.81	17.12	0.6	4.15	433.2	Rosuvastatin (2.5 mg qd)
21st October 2024	21.04	1.31	0.84	16.03	-	-	-	Rosuvastatin (5 mg qd)
26th November 2024	21.58	1.44	0.77	16.2	0.6	3.37	655.7	Rosuvastatin (10 mg qd)
24th December 2024	22	1.16	0.82	16.93	-	-	-	Rosuvastatin (20 mg qd)
14th January 2025	18.56	1.21	0.76	14.3	0.7	3	613	Rosuvastatin (20 mg qd)
25th February 2025	18.04	1.06	0.76	14.17	0.7	3.61	748.3	Rosuvastatin (20 mg qd) + Ezetimibe (10 mg qd)
22nd April 2025	17.55	1.09	0.77	13.54	0.6	3.63	858.2	Rosuvastatin (20 mg qd) + Ezetimibe (10 mg qd)
18th June 2025	14.58	0.98	0.79	12.05	0.6	2.58	939.8	Rosuvastatin (20 mg qd) + Ezetimibe (10 mg qd)
14th October 2025	15.81	1.01	0.87	12.28	0.7	2.75	860.5	Rosuvastatin (20 mg qd) + Ezetimibe (10 mg qd)

## Discussion

FH is a rare inherited metabolic primarily caused by pathogenic mutations in four genes, namely, *LDLR*, *ApoB100*, *PCSK9*, and *LDLRAP1*, which account for approximately 85%–90%, 5%–10%, 1%–3%, and <1% of FH cases, respectively (4). Mutations in the *LDLR* gene are classified into two types based on the degree of loss of LDLR function: null (≤2% LDLR activity) and receptor-defective (2%–70% LDLR activity) variants (4). The clinical severity of the HoFH phenotype is determined by the extent of residual LDLR function, and patients harboring receptor-null variants exhibit higher levels of LDL-C and a poorer prognosis. The study by Bertolini et al. demonstrated that the c.682G > A mutation reduces LDLR activity to 40%–45% of normal levels, classifying it as receptor-defective variant (6). This mutation site is also recognized as one of the most prevalent *LDLR* gene mutation sites among patients with FH in Hong Kong (7).

However, the maternally inherited c.1187-10G > A mutation identified in this study is comparatively rare. This receptor-deficient variant activates an alternative splicing site, leading to aberrant mRNA processing and the subsequent production of a truncated protein (8). Notably, patients harboring this mutation, particularly in true homozygous or compound heterozygous states, often present with extremely early-onset disease, markedly elevated LDL-C levels, and a propensity for rapidly progressive ASCVD. This mutation site was first reported in 2001 in a heterozygous individual of Anglo-Saxon descent (9), with subsequent cases of HeFH documented in France (10), the Philippines (11), and other countries. The first pediatric case of homozygous c.1187-10G > A FH in China was reported in 2015, with the patient developing atherosclerosis at 5 years of age (8). Pediatric patients with cHeFH harboring paternal c.1A > C and maternal c.1187-10G > A mutations were recently reported in India. Similar to our patient, both cases demonstrated a poor response to statin therapy (12). The patients underwent total coronary artery bypass grafting for coronary atherosclerotic heart disease at 9 years of age and received prophylactic liver transplantation to prevent further disease progression. In this case report, the patient presented with cutaneous xanthomas; however, owing to early identification and intervention, no cardiovascular manifestations, such as chest tightness or dyspnea, are currently evident. Follow-up studies will include regular assessments with echocardiography, electrocardiography, and measurement of carotid intima-media thickness. Nevertheless, this case underscores the importance of initiating comprehensive FH evaluations—including LDL-C measurement and genetic screening—for all young children presenting with cutaneous xanthomas, irrespective of age. The findings highlight the importance of early identification and pharmacological intervention in delaying the progression of ASCVD.

Lipid-lowering therapy should be initiated as early as possible following the diagnosis of FH to prevent further escalation of the risk of ASCVD. Lifestyle modification and dietary optimization form the cornerstone of FH management. Current recommendations include reducing saturated fat intake to less than 7% of total daily energy intake, restricting dietary cholesterol to under 200 mg per day, and increasing the consumption of vegetables (13). Additionally, pharmacological therapy should be initiated as early as possible following the diagnosis of FH. Statins represent the first-line therapy for children and adolescents with FH, with high-intensity statins typically recommended for therapeutic management. These agents can reduce LDL-C levels by 23%–40%, and accumulating evidence supports their safety in pediatric populations (14, 15). The currently recommended age for initiating statin therapy in children is 8–10 years (16). However, earlier treatment initiation is advised owing to the significantly elevated LDL-C levels in patients with HoFH. The earliest reported case of statin therapy in a patient with FH was recently reported in Vietnam, where rosuvastatin treatment was initiated at 1.4 years of age (17). A notable feature of the present case is the initiation of rosuvastatin therapy immediately after diagnosis at 2 years and 3 months of age, followed by combination therapy with ezetimibe. The levels of liver and muscle enzymes remained largely within normal ranges over one year of monitoring. To the best of our knowledge, this represents a relatively early and detailed case report of statin initiation in a pediatric patient with HoFH, providing valuable clinical evidence supporting current guideline recommendations. Ezetimibe, the most commonly used non-statin lipid-lowering agent, reduces LDL-C levels and other key lipid and lipoprotein parameters by inhibiting the absorption of intestinal cholesterol. It achieves LDL-C reductions of 13%–20% with a low incidence of adverse events (18). Although treatment with rosuvastatin followed by ezetimibe reduced LDL-C levels in our patient by 26.5% within one year; the levels remained above the recommended target of 3 mmol/L (4). This underscores the need for implementing additional lipid-lowering strategies. In recent years, several novel agents, including PCSK9 inhibitors, bile acid sequestrants, lomitapide, and mipomersen, have been investigated for patients with HoFH. However, the pediatric use of these agents is limited by age restrictions, and several remain unapproved by the FDA for children (19–21).

According to the latest EAS consensus (4), LA should be initiated as early as possible in pediatric patients with FH who exhibit a suboptimal response to drug therapy, provided conditions permit. Ideally, treatment should be initiated before 3 years of age and no later than 8 years to delay the progression of atherosclerosis. However, access to LA for very young children remains limited in many developing countries, representing a key clinical challenge highlighted by this case report. LA remains the most effective acute therapeutic intervention for severe FH, achieving reductions of >70% in serum LDL-C levels per treatment session (16). A recent large-scale international cohort study confirmed that the initiation of lipoprotein plasma exchange in childhood significantly improves cardiovascular outcomes and reduces mortality in patients with HoFH (22).A primary therapeutic limitation in this case was the lack of implementation of LA, resulting in persistently elevated LDL-C levels in the patient. The proposed management plan involves renewed discussions with the family regarding advanced therapeutic options, including LA, followed by a structured surveillance protocol for atherosclerotic complications, incorporating serial cardiac ultrasound, ECG, and scheduled assessment of carotid intima-media thickness.

## Conclusion

This case report describes a rare patient with cHeFH harboring LDLR gene mutations c.682G > A and c.1187-10G > A. This case highlights that cutaneous xanthomas can serve as an important early indicator of FH in infants and young children. Comprehensive lipid profiling and genetic testing should be conducted after detection to confirm a diagnosis of FH. The early initiation and intensification of treatment should be undertaken in patients harboring mutation genotypes associated with extremely high cardiovascular risk. Additionally, should conventional medications prove ineffective, advanced therapies such as LA should be implemented as soon as feasible, accompanied by careful surveillance for cardiovascular complications.

## Data Availability

The original contributions presented in the study are included in the article/Supplementary Material, further inquiries can be directed to the corresponding author.
